# A clinician’s guide to AAV production – How manufacturing platforms shape vector properties

**DOI:** 10.1515/medgen-2025-2024

**Published:** 2025-07-17

**Authors:** Jonas Käsbach, Jørgen Magnus

**Affiliations:** RWTH Aachen University Institute of Biochemical Engineering Forckenbeckstr. 51 52074 Aachen Germany; RWTH Aachen University Institute of Biochemical Engineering Forckenbeckstr. 51 52074 Aachen Germany

**Keywords:** Adeno-associated virus, gene therapy, viral vector manufacturing, production platforms, product identity

## Abstract

Adeno-associated virus (AAV) has become the leading viral vector for *in vivo* gene therapy. This review examines how different manufacturing methods, from mammalian to insect cell-based systems, influence AAV vector characteristics. Though each platform generates vectors with distinct molecular signatures affecting purity, safety and potency, clinical outcomes remain consistent across production platforms. Understanding the nuances of these platforms will still provide valuable insight for clinicians overseeing AAV-based therapy.

## 1 From academic origins to clinical application

Research on AAV production began with quite different ambitions than one would expect looking back at decades of therapeutical development based on this vector. Due to its helper virus dependence and lack of pathogenicity, AAV initially attracted little attention from the medical community when it was identified as a contaminant in adenovirus preparations in 1965 [2, 3, 6]. However, these same characteristics made it a promising candidate for studies on viral latency as well as gene transfer and expression using eukaryotic cells. Almost 20 years later, this research culminated in the cloning of AAV wild-type genomes into bacterial plasmids and the subsequent generation of recombinant AAV vectors, which were first used for the experimental transfer of antibiotic resistance genes into cultured mammalian cells [21, 24, 36, 41]. Production of these early vectors required not only transfection of mammalian cells with plasmids carrying the necessary genes, but due to the helper virus dependency of AAV also their infection with adenovirus. As reviewed in [15], the identification of the necessary adenoviral helper functions during the 1980s and 1990s allowed for the first complete substitution of adenovirus infection by transfection of the functional genes on additional plasmids [12]. Coinciding with this development, the first cell lines were generated in which all the necessary AAV genes as well as the desired transgene were stably integrated [7]. Although this method still required infection with a helper virus, it eliminated the need for complex and expensive plasmid DNA transfections at large production scales.

As demand for preclinical and clinical material increased around the turn of the millennium, emphasis was increasingly placed on improving overall process efficiencies. To this end, an important development was the adaption of a highly productive and suspension-based insect cell protein expression system for AAV manufacturing [43]. This in turn was closely linked to research into first suspension-based production platforms using mammalian cell lines. These approaches have since been enabling the gradual transition from adherent culture vessels to stirred tank reactors, which offer significantly higher volumetric efficiencies and automation capabilities [39]. Irrespective of the cellular production system, this also required more efficient vector purification, which until then had been achieved using laborious ultracentrifugation with toxic density gradients. Novel methods using non-toxic gradient materials as well as affinity and ion exchange chromatography were developed, significantly improving vector recovery and shortening manufacturing timelines [49]. Thirty years after its discovery, the first phase I trial of an AAV gene therapy treating cystic fibrosis was initiated [13]. It would take nearly two more decades before the first AAV-based gene therapies were approved in Europe in 2012 and in the United States in 2017, targeting a fatty acid metabolism disorder and an inherited form of blindness [10, 11].

## 2 Current manufacturing platforms and emerging technologies

Today, research into AAV gene therapy has become highly diverse and includes strategies for reduced immunogenicity, more precise tissue targeting and much more. One focus of this work is capsid engineering through rational design, directed evolution, and, increasingly, machine learning techniques. Rational design involves making structural modifications, such as amino acid substitutions or chemical conjugation, based on an understanding of AAV and host cell biology. Directed evolution, on the other hand, exploits random sequence diversification under selective pressure to identify variants with specific traits [46]. Like the capsid, the AAV genome is also systematically being modified by engineering transgenes, promoters and other regulatory elements. For instance, tissue-specific promoters can be used to restrict expression to target cells and mitigate immune responses to the transgene. A recently published comprehensive overview of the field can be found in [46] and is beyond the scope of this review, which is intended to provide interested members of the medical community with a fundamental understanding of major AAV manufacturing technologies and their potential impact on the properties of the vector product. Nevertheless, a brief outline of AAV vectorology will be given in the following.

Wild-type AAV (wtAAV) particles carry a 4.7 kb single-stranded DNA genome containing 145 bp inverted terminal repeats (ITRs) at both ends. The coding sequence consists mainly of replication (*Rep*) and capsid (*Cap*) genes, with the T-shaped ITRs serving as origins of replication, promoters and genome packaging signals. Measuring 25 nm in diameter, the non-enveloped AAV capsid is assembled from the *Cap*-encoded viral proteins VP1, VP2, and VP3, most commonly in a 1:1:10 ratio. Exposed variable loops within the VP3 sequence dictate interactions with receptors on the host cell surface and thereby determine tissue specificity and immunogenicity of the 13 natural serotypes discovered to date [37, 46]. Following receptor-mediated endocytosis, the low pH of endosomes induces conformational changes in the capsid, facilitating endosomal escape. Once a viral particle has entered the nucleus and its single-stranded genome has been uncoated, it must be converted into double-stranded DNA, a rate-limiting step for transduction and transcription. The resulting genetic element can then undergo ITR-mediated circularisation and either establish episomal latency or rarely integrate into human chromosomes [46]. When a suitable helper virus infects a host cell latently infected with AAV, it provides the essential helper functions E1, E2a, E4 and VA. In their presence, the *Rep* and *Cap* genes native to AAV are expressed, driving genome replication and forming the three VP proteins [37, 45]. Rep proteins also act as packaging signals, by binding primarily to the ITRs of newly synthesised AAV DNA, which is then packaged into preassembled empty capsids [37]. Recent research on the utilization of viral polycistronic mRNA has revealed that the *Cap* locus encodes two other proteins, assembly-activating protein (AAP) and membrane-associated accessory protein (MAAP), whose role in the AAV life cycle is not yet fully understood. While AAP appears to facilitate the assembly of VP proteins into intact capsids in most serotypes, MAAP promotes cellular egress of AAV but negatively affects viral DNA replication, suggesting a role in latency [1, 19]. After reaching titres of around 10^5^ particles per cell, the host cell lyses under the high viral load, releasing infective AAV [6].

The only virus-derived genetic elements that must be retained in recombinant AAV (rAAV) vectors are the ITR sequences that flank the transgene construct and are necessary for genome packaging during production. Because ITRs exhibit only minimal promoter activity in the absence of Rep proteins, functional elements like promoters, enhancers, polyadenylation and localisation signals must be included in the transgene cassette. Usually, the ITR sequences of the well characterised AAV2 are used, which can also be packaged into capsids of other natural or engineered AAV serotypes to change the properties of the vector [37]. However, this strategy must be reassessed for each application, as both production yield and infectivity can vary considerably between the different serotypes. For instance, AAV2 and AAV6 are known to generate significantly lower cell-specific yields than other natural serotypes. This can be attributed to heparin-binding motifs within the capsids of these serotypes, which when deleted lead to significantly higher yields and release of viral particles into the medium [22, 44]. AAV6 can also have a significantly poorer particle-to-infectivity ratio, which indicates further potential for optimization, as this serotype can infect both epithelial cells and cardiomyocytes particularly well *in vitro* [20, 22]. The complete deletion of *Rep* and *Cap* genes from rAAV ensures that they are unable to replicate even upon helper virus infection. Since Rep proteins also mediate site-specific chromosomal integration *in vitro*, the absence of these proteins in rAAV meant that the risk of genomic disruptions was long considered to be negligible. However, recent research has highlighted the possibility of random genomic integration in humans and non-human primates. Although no definitive link has been established between this phenomenon and cellular malignancies, the implications of these findings remain controversial and are under continued investigation [26, 45]. Another difference between wild-type and rAAV is found in their transduction efficiency. While every single wtAAV particle can be infectious in some cases, frequently only one in a hundred rAAV results in gene expression [48]. Consequently, rAAV vectors may need to be administered at higher doses to achieve the desired therapeutic effect, which in turn can result in increased immunogenicity. Some of the factors causing this discrepancy can be attributed to the current manufacturing processes for AAV gene therapies, which will be discussed in more detail below.

A common feature of large-scale rAAV production platforms in use today is that they mostly produce capsids that either do not contain the entirety of the desired DNA cargo or no DNA at all. Mechanistic modelling of various production systems has revealed that the level and timing of vector genome replication often represents the bottleneck in the production of filled capsids. While the expression of all components necessary for virus production seems to be synchronised almost perfectly during infection with wtAAV, in artificial rAAV production the number of empty capsids produced is so high that transgene replication cannot keep up [9, 31, 48]. As a result, state-of-the-art processes usually reach full/empty ratios of only 8 – 30 %, although some recent experimental studies claim to have achieved almost complete capsid saturation [27, 32]. In order to thoroughly characterise rAAV products and manufacturing platforms, complex analytical methods are therefore required, which have been extensively reviewed in [18]. In short, three primary types of viral titres are used for this purpose. The total amount of fully assembled capsids is termed capsid titre, expressed as capsid particles per millilitre (cp/mL) and most commonly determined using enzyme-linked immunosorbent assays (ELISAs). Such methods do not distinguish between empty capsids and those carrying the vector genome. As viral capsids can elicit a strong immune response when administered, this information is important to assess and minimise the risk of adverse reactions. For dosing of systemically administered AAV gene therapies, the number of vector genomes per kilogramme of body weight is used, with the corresponding genome titre stated as vg/mL. Before measurement by quantitative polymerase chain reaction (qPCR), unencapsidated DNA is enzymatically degraded. This value therefore only specifies the amount of vector genomes available for transduction, which is crucial for determining transgene expression levels. By comparing genome and capsid titres of an rAAV sample, full/empty ratios can also be determined. Lastly, *in vitro* assays are used to quantify the actual biological activity of the vector in infectious units per millilitre (IU/mL). As such methods are time-consuming and difficult to automate, they are generally only used in preclinical development [18].

**Figure 1:**
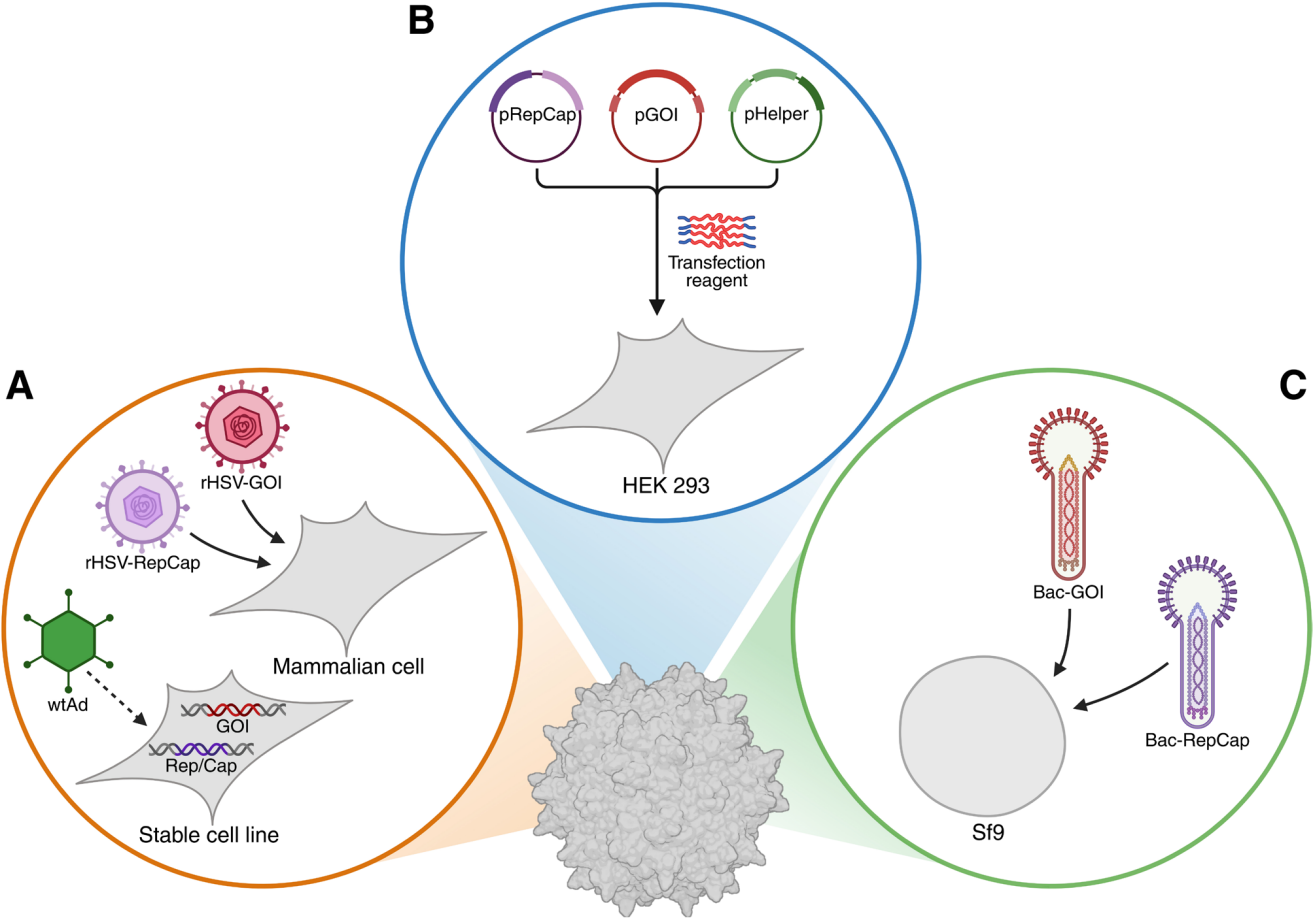
Production platforms for rAAV manufacturing **(A)** Viral infection-based mammalian platform. Two rHSVs are used to deliver *Rep* and *Cap* as well as the GOI. These genes can also be integrated into stable cell lines requiring only helper virus infection with wtAd. Complete integration of all necessary genes has recently been achieved, eliminating the need for viral infection. **(B)** Transient transfection platform. Plasmids carrying the necessary genes form complexes with a transfection reagent and are then transfected into HEK 293 cells. **(C)** Baculovirus expression vector system. Recombinant baculovirus infection supplies Sf9 insect cells with all necessary genes to produce rAAV. Created in BioRender using PDB accession number 7KFR. Magnus, J. (2025) *https://BioRender.com/f96o393.*

Reliable process control has become increasingly important as AAV production has expanded from purely academic to industrial production in scales of up to 2000 L. Today, manufacturers of clinical grade vector material typically employ one of three primary production strategies, as illustrated in Figure 1. The earliest system still in use is based on infecting mammalian cells using suitable viruses and exists in several variations that differ in the way necessary wtAAV genes as well as the gene of interest (GOI) are introduced into the cells. One such method works by co-infecting established human embryonic kidney 293 (HEK 293) or baby hamster kidney (BHK) cell lines with two recombinant herpes simplex viruses (rHSVs). Since rHSV itself can provide helper functions for rAAV production, it is sufficient to generate one such virus to transfer the ITR-flanked GOI into the cells, and another for the necessary *Rep* and *Cap* genes [32, 46]. Although this approach has achieved yields of 10^11^ vg/mL, it is rarely used today [34]. A meta-analysis of the U.S. National Library of Medicine database published in 2022 found that only 3 % of all rAAV vectors used in clinical trials were produced in this way [38]. Stable cell lines represent an evolution of this method, where some of the genetic information required to produce rAAV is stably integrated into the cells, either by plasmid retention using selection markers or by genomic integration. Cells derived from HEK 293, A549 and HeLa lines have primarily been used for this purpose and are categorised as packaging or producer cell lines in this context. Only the serotype-specific *Rep* and *Cap* genes are integrated into packaging cell lines, which therefore require infection with a wild-type helper adenovirus (wtAd) as well as with an Ad carrying the transgene. In producer cell lines on the other hand, the GOI is stably integrated in addition to *Rep* and *Cap*, so only the supply of helper functions by wtAD infection is necessary to induce AAV production. Currently, 8 % of all rAAV vectors for clinical trials are produced using stable cell lines and there is a strong focus on optimizing such systems [38]. Their benefits for large-scale manufacturing are considerable, as they are readily scalable and require only small amounts of plasmid DNA. However, current methods still use infectious viruses and often require extensive cell line development for each desired GOI and rAAV serotype, severely limiting their flexibility. In a recent innovation, a self-silencing adenovirus has thus been constructed that autorepresses the expression of all its genes except the helper functions during rAAV production. This virtually eliminates adenoviral contamination of the product [46]. Moreover, stable integration of all necessary genes under the control of inducible promoters has been demonstrated, completely eliminating the need for helper virus infection. In combination with advanced process engineering, impressive titres of over 10^12^ vg/mL and full/empty ratios of over 30 % have been achieved with this system [8].

The most common rAAV manufacturing method also utilises a mammalian platform through transient transfection of HEK 293 cells. Three plasmids are generally used for this purpose: one encodes the GOI flanked by ITRs, another the *Rep* and *Cap* genes of the desired serotype and a third all necessary helper functions. In order to be taken up by the cells, these plasmids must form complexes with a suitable transfection reagent such as polyethyleneimine (PEI), a delicate step that can determine the success of the whole process. The transient transfection approach can shorten process times, involves no infectious helper virus, offers flexibility in selecting different transgene-capsid combinations and is therefore used for 69 % of all clinical trials [38]. As a mammalian platform, it is also capable of generating human-like post-translational modifications. However, it can suffer from batch-to-batch variations in vector quality due to the sensitive transfection process. In addition, large quantities of clinical-grade plasmid are required for commercial applications, significantly increasing costs. Consequently, research is still being done to optimise all aspects of this system e. g. by developing more efficient PEI alternatives, and titres of over 10^11^ vg/mL have been achieved. At the molecular level, it has evolved into a dual plasmid system by combining *Rep*, *Cap* and adenoviral helper genes on a single plasmid, simplifying the protocol while maintaining versatility for serotype and GOI swapping. Another recent innovation has combined all components into a single plasmid, resulting in higher yields and lower batch-to-batch variability [32, 34, 46].

As a popular alternative to methods based on mammalian cells, the well-established baculovirus expression vector system (BEVS) has been adapted for rAAV production and now accounts for 20 % of all rAAV vectors used in clinical studies [38]. In this approach, recombinant baculoviruses are used to introduce the necessary genes into cells of the fall armyworm *Spodoptera frugiperda*, often using the Sf9 lineage. While the first BEVS generation required separate delivery of *Rep* and *Cap* genes as well as the GOI and thus co-infection of Sf9 cells with three recombinant baculoviruses, the system was simplified by combining *Rep* and *Cap* into one cassette. Further progress was made in the development of Sf9 cells that stably express *Rep* and *Cap*, thereby requiring only one transgene-carrying baculovirus and significantly increasing yields [43, 46]. The BEVS technique was originally developed because, unlike mammalian cells, Sf9 cells could grow in suspension at high cell densities. While this advantage has become less pronounced due to the adaptation of mammalian cells to serum-free suspension culture, BEVS production still offers very high titres of up to 10^12^ vg/mL and significantly improved full/empty ratios [27]. Moreover, although infectious viruses are used in this system, residual baculoviruses are generally considered to be less efficient in transducing human cells than HSV or Ad and thus pose a lower risk to safety. One of the challenges hindering a more widespread adoption of BEVS is the inherent genetic instability of baculoviruses, which may lead to defective rAAV particles. In addition, deviations from wtAAV capsid protein stoichiometry and post-translational modification have been demonstrated for BEVS-derived rAAV [46]. The extent to which such process and product characteristics may affect safety and efficacy of rAAV formulations is subject to ongoing research and will be addressed in the following.

## 3 Production platform fingerprints in AAV gene therapy vectors

**Figure 2:**
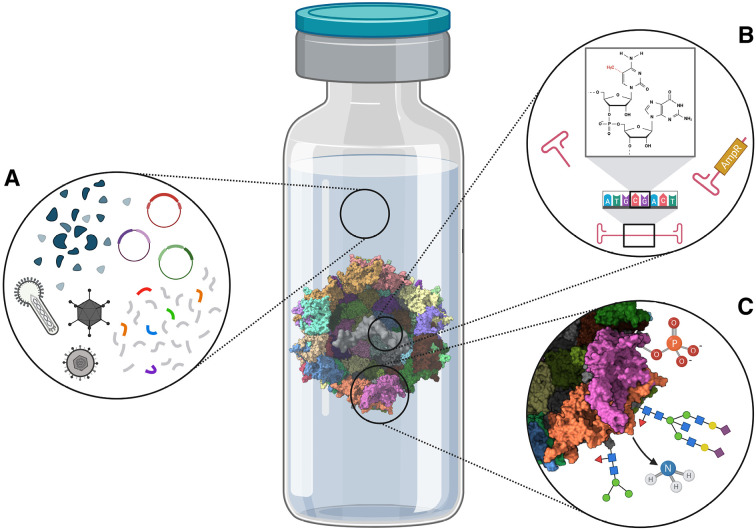
Process and product-related impurities in therapeutic rAAV formulations **(A)** Process-related impurities. These include HCD, HCP, residual virus and residual plasmid DNA. **(B)** DNA impurities and modifications. Unmethylated CpG motifs, truncated ITR fragments and reverse packaging of antibiotic resistance genes can pose serious risks to safety. **(C)** PTMs of capsid proteins. Significant effects of small molecule modifications like phosphorylation and deamidation have been described. Glycosylation patterns also differ between rAAVs produced in insect and mammalian cells. Created in BioRender using PDB accession numbers 7L5U and 1D28. Magnus, J. (2025) *https://BioRender.com/d93i955.* Molecular graphics and analyses performed with UCSF ChimeraX [29].

Although the release of clinical grade material derived from human or animal cell lines has long been subject to strict regulatory control, the identification and reduction of process and product-related impurities in therapeutic rAAV formulations poses a particular challenge. A graphical representation of the product variants described hereafter is shown in Figure 2. While the infectious helper viruses used in some production processes of course represent inherent contaminants, specific clearance limits have not yet been established [23]. One reason for this is that these are usually distinct enough from rAAV to be separated during purification. Enveloped viruses such as baculoviruses or HSV are often disrupted by detergent lysis, while Ad capsids, which are thermally labile compared to rAAV, can be degraded at 51 – 56 °C. As AAV is one of the smallest animal viruses, most other viruses can also be removed by nanofiltration. Remaining viral impurities are further reduced during rAAV purification by chromatography or ultracentrifugation [32]. Other types of impurities and their effects have only recently come under investigation and are rarely compared between identically treated vector preparations from different production systems. For instance, residual viral, plasmid or host cell DNA (HCD) from the manufacturing processes may not be sufficiently separated or even be packaged in rAAV capsids, making the currently accepted limit of < 10 ng HCD per dose difficult to achieve [42]. Thus, before endonuclease treatment was commonly implemented to degrade unencapsidated nucleic acids after cell lysis, up to 350 ng residual HCD from BHK cells and 6.5 µg HSV DNA per dose were administered in a phase 2 clinical trial [14, 32]. When HEK 293 cells are used, HCD is of particular concern as they express the adenoviral E1 protein as a result of immortalisation, which can inactivate the cellular tumor suppressor p53 and therefore have an oncogenic effect [42]. Furthermore, a recent study has revealed unencapsidated host cell microRNA in HEK 293 and BEVS-derived rAAV vectors with the latter containing higher concentrations. miRNAs are part of a group of small non-coding RNAs and can induce translational repression and degradation of mRNA, which in certain cases has an oncogenic effect [33]. Additional research has demonstrated that rAAV vectors can induce lot-specific, but not production platform-specific innate immune signalling and thus trigger inflammatory cytokine responses [5]. As these impurities can be eliminated more easily, research today focuses mainly on characterising and minimising packaging of non-transgenic DNA into rAAV capsids.

Making up less than 0.2 %, HCD, helper or *Rep*/*Cap* sequences represent the minority of these encapsidated DNA impurities both in HEK 293 transfection and BEVS-derived preparations. This is due to the fact that the mechanism of such defects appears to be reverse packaging initiated from ITR sequences, resulting in up to 1 % of the packaged sequences originating from the GOI-encoding plasmid or baculovirus [32, 40]. Such sequences can be particularly hazardous if they contain the antibiotic resistance genes that are necessary for bacterial plasmid production or the selection of recombinant viruses. In one example from literature, copies of the ampicillin resistance gene accounted for up to 6 % of the packaged vector genomes generated in mammalian stable cell lines. Strategies to minimise such risks include the use of enzymatically produced DNA for transfection, as well as a backbone design in which these genes are cloned sufficiently separated from the ITR sequences [32]. Another recent discovery has revealed that the capsids identified as empty by qPCR are mostly not empty, but contain truncated or unresolved ITR fragments. Since ITRs contain CpG motifs, i. e. a cytosine followed by a guanine base, this impurity also has potential implications for the safety of rAAV vectors. Unmethylated CpG dinucleotides can trigger the innate immune response and thereby induce adverse reactions [40, 42]. Such epigenetic modifications are not to be neglected even in intact vector genomes. In a landmark study, in which HEK 293 transfection and BEVS-derived rAAV were directly and independently compared for the first time, differences in genome methylation patterns were detected between these two main production platforms. Two known repressive methylations in the promoter as well as in the polyadenylation signal were significantly increased in BEVS-derived rAAV, while an activating intragenic methylation appeared higher in rAAV from HEK 293 cells. This may well contribute to findings that rAAV produced in human cells for this study were significantly more potent than BEVS-derived vectors in several cell types *in vitro* as well as in different mouse tissues and even in the liver of humanized mice *in vivo* [35]. Although the BEVS system has since been further refined and in some studies even produces more potent vectors and less impurities, a second independent study was able to confirm these comprehensive results [16, 25, 30].

Viral and host cell proteins (HCP) are another type of impurity that can usually be readily separated during purification. Nevertheless, HCPs in particular are part of detailed analytical panels, as they are sometimes copurified with full rAAV capsids and can have production platform-specific effects. Early vectors produced in stable cell lines were still contaminated with Ad proteins when purified by ultracentrifugation. Ion exchange chromatography can achieve better separation and thus reduce their strong immunogenic effect. Some clinical preparations generated using HSV infection were also found to contain larger amounts of viral protein, but so far no negative effect has been observed [32]. The influence of impurities from the BEVS system was further investigated in the head-to-head comparison study by spiking a purified fraction of empty capsids and insect cell HCPs into a preparation of filled capsids. Significant loss of transgene expression was observed, whereas spike-ins with human-produced empty capsids and HCPs had no effect. This effect could in part be due to divergent post-translational modifications (PTMs), since N-linked glycans were identified on insect cell-derived HCPs that deviate from the human glycosylation pattern [35].

PTMs have long been part of the generally accepted critical quality attributes in the production of therapeutic proteins such as monoclonal antibodies. Although they are not yet part of standardised lot release panels for rAAV vectors, they are a growing focus of research in this area [42]. While an early study on vectors produced in HeLa cells did not detect any glycosylation, different PTMs have now been identified on HEK 293 and BEVS-derived capsids. Of particular relevance is the fact that the latter contain more PTMs in the entire capsid sequence and especially in an rAAV8 cell surface receptor binding domain [35]. Overall, research on the type and number of O- and N-linked glycans is still inconclusive. However, a surprisingly high number of high-mannose glycans and, in contrast, few sialylated glycans have recently been identified on rAAV capsids, which aligns well with the known requirements for virus-host cell interactions [47]. Smaller PTMs include phosphorylation, which can significantly reduce transgene expression, and especially deamidation, where the amide group of an asparagine side chain is removed by reacting with an adjacent amino acid [28, 42]. This modification can lead to a differential T cell response and progressive loss of infectivity. As a result, rAAV processing and storage times could significantly affect their potency [4, 17].

In spite of the differences between the various rAAV manufacturing systems discussed in this review, no statistically significant variations in safety, efficacy and durability of transgene expression have been observed [38]. Due to their unique advantages, suited to the various stages of gene therapy development from the preclinical phase to commercialisation, they are likely to coexist for the foreseeable future. Medical professionals will therefore benefit from an awareness of the risks and opportunities of rAAV production platforms.
